# Analysis of the modified rankin scale in randomised controlled trials in acute stroke: a systematic review

**DOI:** 10.1186/1745-6215-16-S2-P130

**Published:** 2015-11-16

**Authors:** Aimie Nunn, Laura Gray

**Affiliations:** 1 MRC Clinical Trials Unit, University College London, London, UK; 2 Department of Health Sciences, University of Leicester, Leicester, UK; 3 Leicester Clinical Trials Unit, University of Leicester, Leicester, UK

## Background

Historically, trials in acute stroke have largely been unable to show benefit of new interventions. Trials have previously favoured dichotomous analysis of outcome measures employing an ordinal scale, such as the Modified Rankin Scale (mRS). In 2007, the OAST Collaboration showed that preserving the ordinal nature of these scales increased statistical power, recommending the use of ordinal logistic regression where proportional odds could be assumed. A systematic review of trials and protocols published since 2007 was conducted to re-evaluate statistical methods used and assess whether practice has changed.

## Methods

Searches of electronic databases identified trials published between Jan 2007 and July 2014 in acute ischaemic stroke using an ordinal measure of dependency as the primary outcome. Published protocols were also identified to evaluate proposed statistical analyses.

## Results

Forty-two RCT results publications were identified. The majority of studies used a dichotomous analysis (25, 59.5%), eight (21.4%) retained the ordinal scale and nine (19.0%) used another type of analysis (two of which used a sliding dichotomy). Sixteen published protocols were identified, nine (56.3%) of which intend to use an ordinal method of analysis. Six (37.5%) intend to use a dichotomous analysis and one trial a sliding dichotomy (6.3%).

## Conclusions

Trials published since 2007 still favour dichotomous analyses over ordinal. Assessment of ongoing trial protocols shows that ordinal analyses are being incorporated more often, although trials with a published protocol may reflect a biased sample of all trials. Stroke trials, where appropriate, should retain the ordinal nature of dependency scales.

